# 

**Published:** 1893-11-18

**Authors:** 


PRICE TWOPENCE.
No. 373, Vol. XV.—Nov. 18th, 1893.
[Registered at the General Post Office as a Newspaper for transmission in the United Kingdom and Abroad.]

WITH EXTRA NURSING SUPPLEMEN[illegible]

Per Annum, 8s. 6d., post free.

Monthly Parts, 6d.; post free, 8d.

Ditto per Annum, 8s., post free.

LIBRAR[illegible] Index S. G. O[illegible]

### THE HOSPITAL

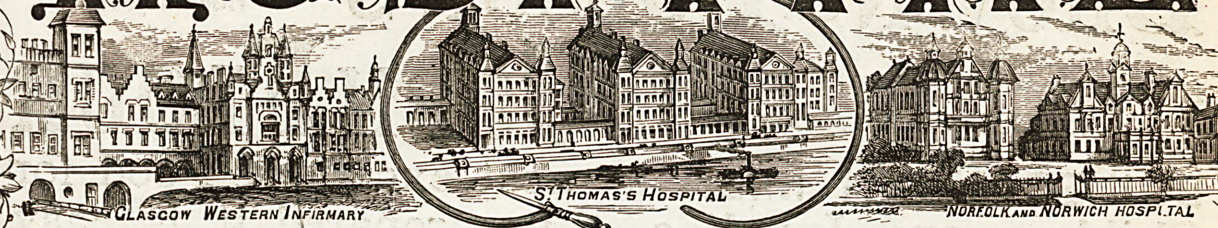

WITH EXTRA NURSING SUPPLEMENT.

No. 373. Vol. XV. Saturday, November 18th, 1893. [Twopence.

